# Supplementary material for: CoCl2-Mimicked Endothelial Cell Hypoxia Induces Nucleotide Depletion and Functional Impairment That Is Reversed by Nucleotide Precursors
Source: Biomedicines. 2022 Jun 28;10(7):1540. doi: 10.3390/biomedicines10071540 (PMC9313011; doi:10.3390/biomedicines10071540)
Supplement: Supplementary file 1 [file biomedicines-10-01540-s001.zip › biomedicines-1766312-supplementary.pdf]

Supplementary Material

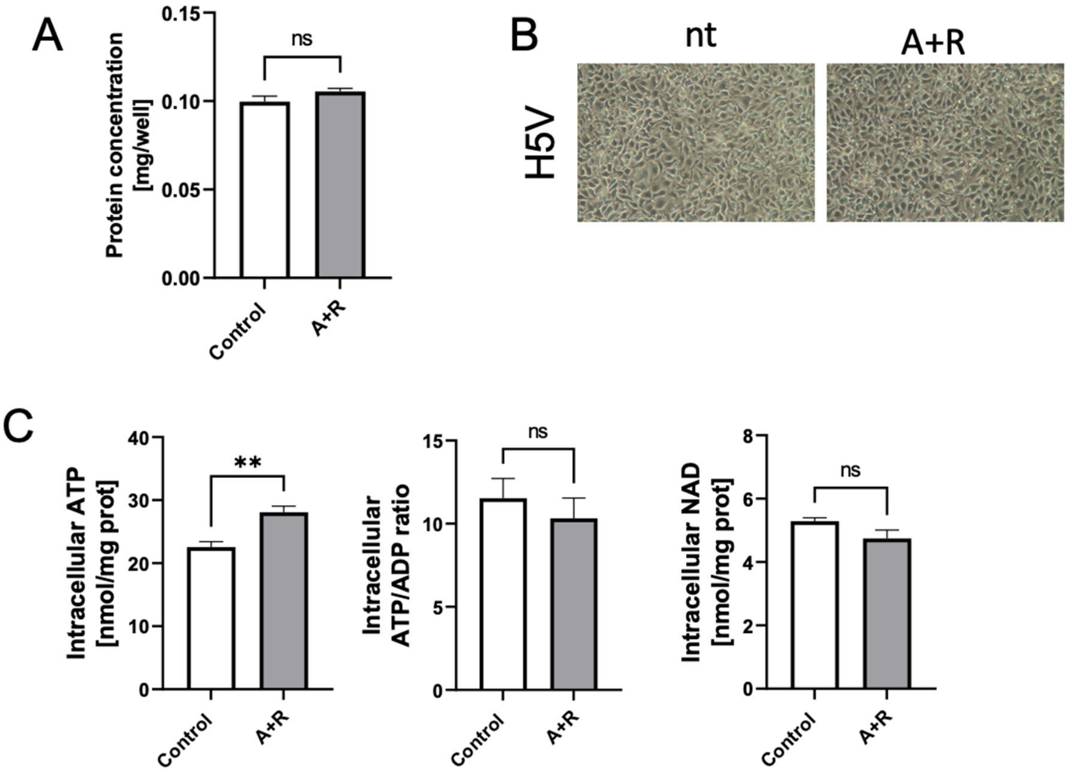

Figure S1. The effect of adenine and ribose supplementation on intracellular nucleotide status in non-stimulated H5V cells. A. Total cell protein, B. Representative H5V cell images after treatment with 100 μM adenine and 2.5 μM ribose (A+R); C. Intracellular nucleotide concentration after A+R treatment. Results are shown as mean ± SEM; *n*=6; $^{**}p<0.01$.
